# Preoperative neoadjuvant chemotherapy using nanoparticle albumin-bound paclitaxel followed by epirubicin and cyclophosphamide for operable breast cancer: a multicenter phase II trial

**DOI:** 10.1007/s12282-016-0748-6

**Published:** 2017-01-03

**Authors:** Manabu Futamura, Yasuko Nagao, Kazuhiro Ishihara, Makoto Takeuchi, Takumi Nakada, Yoshihiro Kawaguchi, Masayoshi Asano, Iwao Kumazawa, Takashi Shiroko, Kasumi Morimitsu, Ryutaro Mori, Masahito Nawa, Toshio Shimokawa, Kazuhiro Yoshida

**Affiliations:** 1 Department of Surgical Oncology, Graduate School of Medicine, Gifu University, Gifu, Japan; 2 Department of Breast Surgery, Gifu Prefectural General Medical Center, Gifu, Japan; 3 Department of Surgery, Gihoku Kosei Hospital, Gifu, Japan; 4 Department of Breast Surgery, Kizawa Memorial Hospital, Minokamo, Japan; 5 Department of Breast Surgery, Gifu Municipal Hospital, Gifu, Japan; 6 Department of Breast Surgery, Murakami Memorial Hospital, Gifu, Japan; 7 Department of Surgery, Municipal Ena Hospital, Ena, Japan; 8 Department of Surgery, Ibi Kosei Hospital, Gifu, Japan; 9 Department of Surgery, Takayama Red Cross Hospital, Takayama, Japan; 10 Department of Regional Medicine, Graduate School of Medicine, Gifu University, Gifu, Japan; 11 Clinical Study Support Center, Wakayama Medical University, Wakayama, Japan

**Keywords:** Neoadjuvant chemotherapy, Nab-PTX, pCR, SPARC

## Abstract

**Background:**

Recently, the use of taxane-based regimens before anthracycline-based regimens has been shown to achieve high pathological complete response (pCR) rates in patients with breast cancer. Nanoparticle albumin-bound paclitaxel (nab-PTX) has been reported as highly effective and less toxic compared with Cremophor-based Taxol. This phase II clinical trial evaluated the safety and efficacy of preoperative neoadjuvant chemotherapy (NAC) with nab-PTX followed by an epirubicin plus cyclophosphamide (EC)-based regimen for operable breast cancer.

**Patients and methods:**

From June 2012 to January 2014, four cycles of every-3-week (q3w) nab-PTX [plus q3w trastuzumab in cases of human epidermal growth factor 2 (HER2) positivity] followed by four cycles of q3w EC were administered to patients with operable breast cancer (stage IC–IIIA). The primary endpoint was the pCR rate (ypT0/TisypN0).

**Results:**

A total of 55 patients were enrolled, 54 of whom received at least one nab-PTX dose. All patients underwent radical surgery after chemotherapy. The overall pCR rate was 22.2% (*p* = 0.006). The pCR rates for patients with the luminal B, luminal/HER2, HER2-rich, and triple-negative breast cancer subtypes were 10.5, 29.4, 60, and 15.4%, respectively. Stepwise logistic regression analysis revealed only HER2 as a significant factor for pCR (odds ratio 5.603; *p* = 0.024). The expression of secreted protein acidic and rich in cysteine showed no association with pCR. The clinical response rate was 70.4% (38/54), and the safety profile was tolerable.

**Conclusion:**

Preoperative NAC with nab-PTX followed by EC is effective and safe for operable breast cancer.

## Introduction

Of late, NAC has been widely used for locally advanced and early breast cancer, with the purpose of not only downstaging for breast-conserving surgery (BCS), but also chemosensitivity testing in vivo, because a pCR can result in a good long-term prognosis [1]. Therefore, various regimens have been tried and reported to yield better pCR rates.

Since initial reports by the National Surgical Adjuvant Breast and Bowel Project protocol 18 (NSABP-B18), anthracycline-based regimens [doxorubicin (Adriamycin) and cyclophosphamide] have been shown to be effective [2]. Furthermore, additional taxane-based regimens for breast cancer were found to significantly increase the proportion of patients achieving a pCR (26.1%) compared with preoperative anthracycline-based regimens alone (13.7%) [3, 4]. These findings have led to the widespread use of anthracycline- and taxane-based regimens [5]. In Japan, two clinical trials of FEC (5-FU, epirubicin, and cyclophosphamide) and docetaxel demonstrated better results, with a near pCR [quasi-pathological complete response (QpCR); few residual cancer cells] rate of 25–29% [6, 7]. Furthermore, the subtype concept based on biological testing has promoted combination therapy with anti-cancerous drug and molecular target agents like TZ, which is inevitably important for HER2-positive breast cancer and results in high pCR rates [8].

Nab-PTX (Abraxane^®^) is an albumin-bound, 130-nm particle form of paclitaxel, which is a novel taxane formulation that was developed to avoid Cremophor/ethanol-associated toxicities such as peripheral neuropathy and allergic reactions [9]. Although Cremophor-based paclitaxel (Taxol) plays an important role in breast cancer therapy, nab-PTX has been reported as safe and effective for metastatic breast cancer (MBC) in several clinical trials [10, 11]. Recently, several reports using nab-PTX in neoadjuvant settings have been reported [12–15]. Most of these studies involved anthracycline-based regimens followed by nab-PTX. However, Earl et al. [16] demonstrated the effectiveness of administering taxane-based regimens before anthracycline-based regimens. Also, few studies have assessed the adverse events (AEs) of nab-PTX in chemotherapy-naïve patients. From these perspectives, we conducted the present multicenter, single-arm phase II trial to evaluate the efficacy and safety of preoperative NAC with nab-PTX followed by EC for operable breast cancer [Perpetual Study estimated-by United Sections in Gifu for Breast Cancer 01 (PerSeUS BC01)].

## Patients and methods

### Patients

The present study was a multicenter, prospective, open-label, single-arm, phase II clinical trial that recruited patients via central registration. Women aged 20–70 years with histologically proven operable breast cancer (T1c-T3N0-2M0, stage I–IIIB) were enrolled. Patients with a history of previous therapy, including chemotherapy, radiotherapy, hormonal therapy, and immunotherapy, were excluded. All tumors were locally tested for estrogen receptor (ER), progesterone receptor (PgR), HER2, and Ki67 by immunohistochemistry (IHC). Tumors with ≥1% positively stained tumor cells were classified as positive for ER and PgR. HER2 positivity was defined by an IHC score of 3+ or 2+ with gene amplification (>2.0) in fluorescent in situ hybridization (FISH). Patients with different subtypes, including hormone receptor (HR)+/HER2− (luminal B), HR+/HER2+ (luminal/HER2), HR−/HER2+ (HER2-rich), and HR−/HER2− [triple-negative breast cancer (TNBC)] subtypes, were considered eligible. The luminal B subtype was defined by ER or PgR positivity, HER2 negativity, and Ki67 ≥ 15% or a nuclear grade (NG) of 3 [17]. Patients with inflammatory breast cancer, bilateral cancer, mucinous carcinoma, and luminal A subtype were excluded. Axillary lymph node involvement was determined by fine-needle aspiration biopsy in cases of clinically positive nodes or sentinel lymph node biopsy in cases of clinically positive nodes before treatment. Pregnant or lactating women were excluded. Eastern Cooperative Oncology Group performance status (ECOG-PS) of all patients was 0 or 1, and all patients exhibited adequate organ function [aspartate transaminase, alanine transaminase, and bilirubin ≤2.5 times the upper limit of normal, leukocyte count ≥3000/mm^3^, neutrophil count ≥1500/mm^3^, thrombocyte count ≥1 × 10^5^/mm^3^, hemoglobin ≥9 g/dl, creatinine ≤1.5 mg/dl, and normal left ventricular ejection fraction (LVEF) ≥50%]. Patients with active malignancy; active infection; and serious concomitant diseases such as heart failure, diabetes, liver failure, uncontrollable peripheral neuropathy, and/or severe drug allergy were excluded.

The study (UMIN 000009035) was conducted in accordance with the Declaration of Helsinki and was approved by the local ethics committee or review board of each participating institution. All patients provided written informed consent for participation.

### Treatment

**Fig. 1:**
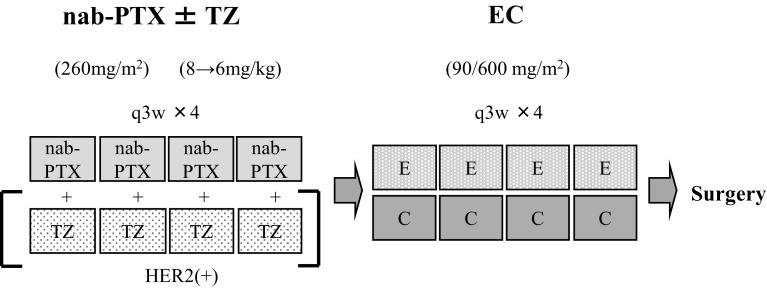
Schema for the study design. *nab*-*PTX* nanoparticle albumin-bound paclitaxel, *TZ* trastuzumab, *E* epirubicin, *C* cyclophosphamide

The study design is shown in Fig. 1. Patients with the luminal B or TNBC subtype received four cycles of every-3-week (q3w) nab-PTX 260 mg/m^2^ followed by four cycles of q3w EC (E: 90 mg/m^2^ and C: 600 mg/m^2^). For HER2-positive patients (HER2-rich or luminal/HER2), TZ 6 mg/kg (8 mg/kg as the loading dose) was administered in combination with nab-PTX. Toxicities were evaluated by National Cancer Institute Common Terminology Criteria for Adverse Events (CTCAE) version 4.0. Each treatment was withheld for a maximum of 3 weeks only in cases of severe toxicity. The dose of chemotherapeutic agents (EC, nab-PTX) could be tapered when febrile neutropenia (FN), grade 3–4 thrombocytopenia, or grade 3–4 nonhematological toxicities (except nausea/vomiting and fatigue) were observed. The first permitted dose reduction was as follows: nab-PTX, 260–220 mg/m^2^ and EC, 90/600–70/450 mg/m^2^. A second dose reduction was permitted if severe AEs occurred after the first dose reduction and was as follows: nab-PTX, 180 mg/m^2^ and EC, 60/400 mg/m^2^. The TZ dose was not tapered in any patients except those with cardiac dysfunction. Prophylactic granulocyte-colony stimulating factor (G-CSG) was not allowed. However, in case of FN, G-CSF was allowed depending on physician’s decision. Relative total dose intensity (RTDI) was shown by the ratio of actual total dose intensity (ATDI) to planned total dose intensity (PTDI) [18].

### Response and toxicity assessments

The primary endpoint was the pCR rate, defined as no histological evidence of residual invasive tumor cells in the breast and axillary lymph nodes (ypT0/TisypN0). The secondary endpoints were the clinical response rate (RR), histological assessment [19, 20], breast-conserving rate, and safety. The clinical tumor response was assessed by the Response Evaluation Criteria in Solid Tumors (RECIST) version 1.1 using computed tomography (CT) or magnetic resonance imaging (MRI) [21]. Patients were considered responders if they achieved a CR or a partial response (PR), which was shown as the clinical RR. A pathological response was defined as a pCR or the presence of minimal residual invasive disease only in the breast. BCS was recorded when lumpectomy, segmentectomy, or quadrantectomy was the final surgical procedure. All patients who received chemotherapy (more than one cycle of each regimen) were evaluated for safety. Laboratory and nonlaboratory toxicities were evaluated using CTCAE version 4.0 [22].

### IHC for secreted protein acidic and rich in cysteine (SPARC)

In the present trial, nab-PTX was initiated before EC. To investigate the association between the efficacy of nab-PTX and the expression of SPARC, we performed IHC for SPARC expression using biopsy samples. A Dako LSAB Kit (Dako, Carpinteria, CA, USA) was used for immunohistochemical analysis. In brief, sections were pretreated by CC1 (Roche, Basel, Switzerland) and incubated with the primary antibody against SPARC (AF941, 1:1000, R&D, Minneapolis, MN, USA) for 1 h at 25 °C. Then, the sections were incubated with biotinylated anti-goat IgG and peroxidase-labeled streptavidin for 10 min each. Staining was completed with substrate–chromogen solution followed by counterstaining with 0.1% hematoxylin. The slides were scored for SPARC expression using a scale of 0–3, where 0 represented negativity and 3 represented strong positivity. We considered SPARC negativity in patients with a score of 0–1 and positivity in patients with a score of 2–3. Scores were subjectively assigned by experienced pathologists.

### Statistical analysis

In previous studies conducted in neoadjuvant settings, the pCR rate for EC followed by docetaxel was 31.3% [5], while that for EC only was 11.0% [23]. The required sample size was estimated based on a threshold pCR rate of 10% and an expected pCR rate of 25%, 90% power, and an alpha error of 0.05 (one-sided) using the binomial test. Given 5% of ineligible patients, the target sample size was determined to be at least 52 patients. Furthermore, to evaluate exploratory variables (age, nodal metastasis, ER, PgR, HER2, and SPARC expression) for pCR, multiple logistic regression analysis with backward stepwise variable selection using Akaike’s information criteria (AIC) was performed [24]. All statistical analyses were performed using R, version 3.2.3. Fisher’s exact test was performed to evaluate relationship between SPARC and pCR.

## Results

### Patient characteristics

Between June 2012 and January 2014, 55 eligible patients with operable breast cancer were enrolled in this trial. The baseline patient characteristics are summarized in Table 1. The median age of patients was 54 years (range 27–69 years). All patients were diagnosed with invasive ductal carcinoma through core needle or vacuum-assisted biopsy. Axillary lymph node metastasis was identified in 33 patients (61.8%). There were five patients (9.1%) with stage I disease, 23 (41.8%) with stage IIA disease, 24 (43.6%) with stage IIB disease, two (3.6%) with stage IIIA disease, and one (1.8%) with stage IIIB disease. ER and PgR positivity was observed in 36 (65.5%) and 31 patients (56.4%), respectively, HER2 positivity in 20 (36.4%), and SPARC positivity in ten (18%). IHC revealed the luminal subtype (HR+/HER2−) in 19 patients (34.5%), luminal/HER2 subtype (HR+/HER2+) in 17 (30.9%), HER2-rich subtype (HR−/HER2+) in five (9.1%), and TNBC subtype (HR−/HER2−) in 14 (25.5%).

**Table 1:** Patient characteristics

	Number of patients	%
Age (years)		
Median	54	
Range	27–69	
≥50	22	40
<50	33	60
Performance status = 0, 1	55	100
Clinical tumor stage		
T1	12	21.8
T2	41	74.6
T3	1	1.8
T4	1	1.8
Clinical nodal stage		
N0	21	38.2
N1	33	60.0
N2	1	1.8
Clinical stage		
I	5	9.1
IIA	23	41.8
IIB	24	43.6
IIIA	2	3.6
IIIB	1	1.8
ER status		
Positive	36	65.5
Negative	19	34.5
PgR status		
Positive	31	56.4
Negative	24	43.6
HER2 status		
Positive	20	36.4
Negative	35	63.7
SPARC expression		
Positive	10	18.9
Negative	43	81.1
Not examined	2	
Subtype		
HR+/HER2− (luminal B)	19	34.5
HR+/HER2+ (luminal-HER2)	17	30.9
HR−/HER2+ (HER2-rich)	5	9.1
HR−/HER2− (triple negative)	14	25.5

*ER* estrogen receptor, *PgR* progesterone receptor, *SPARC* secreted protein acidic and rich in cysteine, *HR* hormone receptor

### Compliance and study completion

Fifty-five patients were enrolled in this study. One patient withdrew before receiving the study drug, and 54 patients who received at least one cycle of nab-PTX were included in the safety and response analysis. Of 54 patients, 51 (94.4%) completed all four cycles. Two patients discontinued nab-PTX because of progressive disease (PD) and one because of toxicity (rash). Dose reduction was required in six (7.4%), including two with neutropenia (grade 4), one with obesity and myalgia. Dose delay (≥1 week) was required in six (11.1%), including two with liver dysfunction, one with cystitis and three with patient’s preference, respectively. Fifty patients (92.6%) completed four cycles of nab-PTX. Furthermore, the study was discontinued in two patients with PD or AST/ALT increase after completion of all nab-PTX cycles.

Consequently, 50 patients received EC, 46 of whom completed the EC regimen (92%). Four patients, including three with PD and one with AEs (an AST/ALT increase after two cycles), were excluded. Dose reduction was required in five patients (10%) due to grade 4 neutropenia. Dose delay was required in 16 patients (32%), including 12 with myelosuppression, one with fatigue, one with inflammation, one with fatigue, and one due to hospital closed. Finally, 46 of 54 (85.2%) completed the whole regimen. RDIs for nab-PTX and epirubicin were 83.7 and 28.3 mg/m^2^/w, and RTDIs for nab-PTX and epirubicin were 96.5 and 94.8%, respectively. All patients underwent curative surgery after chemotherapy.

### Clinical and pathological assessments

**Fig. 2:**
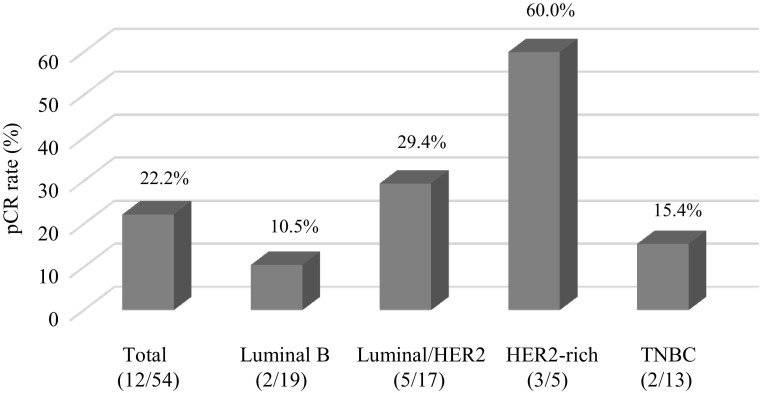
Pathological complete response (pCR) rate for each subtype of breast cancer

**Fig. 3:**
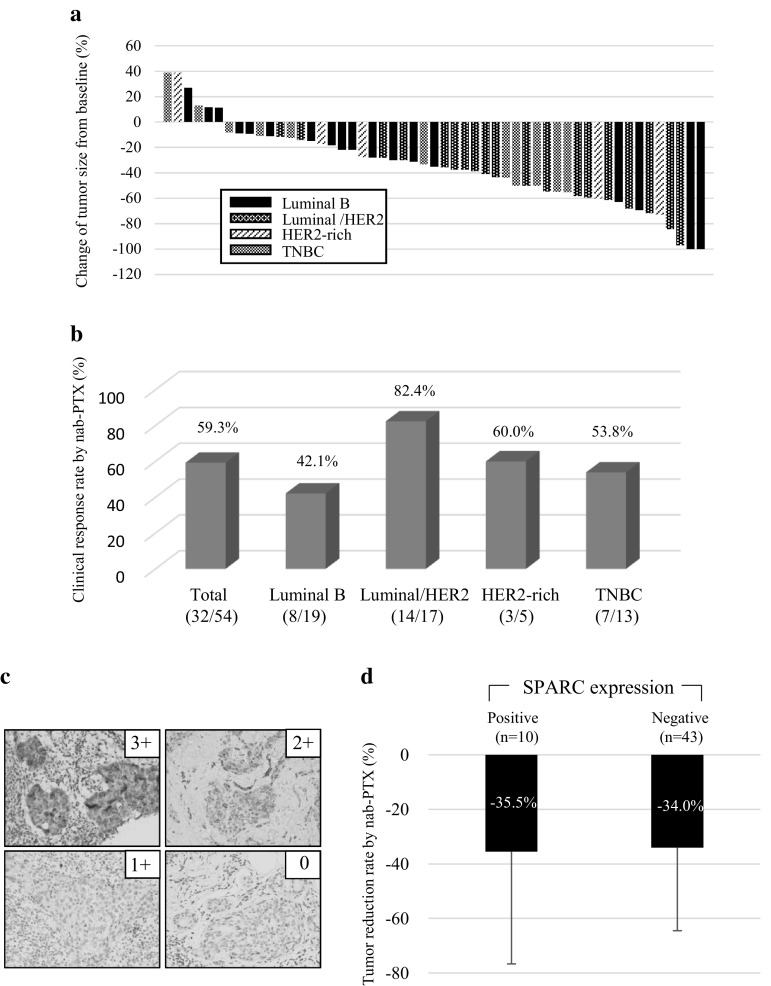
Response to nanoparticle albumin-bound paclitaxel (nab-PTX) therapy. **a** Waterfall plot to show the efficacy of nab-PTX therapy. **b** Clinical response rate after nab-PTX therapy. Each subtype is indicated. **c** Immunohistochemistry for PARC expression. Representative cases are indicated. Scores 0 and +1 indicate negativity, and 2+ and 3+ indicate positivity. **d** The tumor shrinkage rate depending on SPARC expression in tumor. The average rate with an error bar for the standard division is shown

A pCR (ypT0/Tis ypN0, grade 3), which was the primary end point of this study, was observed in 12 of the 54 patients [22.2%; 95% confidence interval (CI) 12.0–35.6; *p* = 0.006]. The pCR rate for patients with the luminal, luminal/HER2, HER2-rich, and TNBC subtypes was 10.5% (2/19), 29.4% (5/17), 60.0% (3/5), and 16.7%, respectively (Fig. 2). In addition, two cases of grade 2b which means extremely marked response by JBCS [19, 20] were observed, indicating that quasi-pCR (QpCR) rate (grade 3 + 2b) [6] was 25.9% (14/54), and the clinical RR was 70.4% (38/54).

nab-PTX resulted in tumor shrinkage in 88.7% patients (except one unevaluable patient), with a clinical RR of 59.3% [(32/54), Fig. 3a, b], while the rate in HER2-positive patients was 77.2% [17/22; luminal/HER2, 82.4% (14/17); HER2-rich, 60% (3/5)] with TZ treatment (Fig. 3a, b). The clinical RR after completion of EC increased to 70.4% (38/54), and it was 81.8% (18/22) for HER2-positive cases [luminal/HER2, 82.4% (14/17); HER2-rich, 60% (3/5)], 73.7% (14/19) for the luminal subtype, and 46.2% (6/13) for the TNBC subtype. Of the patients with pCR, two achieved clinical CR (cCR) after nab-PTX. Of the total number of patients, seven (13.0%) exhibited PD (luminal: 1, luminal/HER2: 2, HER2-rich: 1, and TNBC: 3). Three patients (luminal: 1, HER2-rich: 1, and TNBC: 1) showed PD during nab-PTX therapy and underwent conversion to surgery. BCS was performed for 22 of the 54 (40.7%) patients.

### Safety profile

The incidence of treatment-related AEs (all grades and grade ≥3) is shown in Table 2. During nab-PTX therapy, ≥grade 3 hematological toxicities included neutropenia (48.2%), leukopenia (13%), and an ASL/ALT increase (5.6%), while ≥grade 3 nonhematological toxicities included arthralgia (14.8%), myalgia (13%), peripheral sensory neuropathy (7.4%), peripheral motor neuropathy (1.9%), and rash (1.9%). During EC therapy, ≥grade 3 hematological toxicities included neutropenia (61%), leukopenia (37%), FN (6.1%), anemia (2%), and an ASL/ALT increase (2%), while ≥grade 3 nonhematological toxicities included nausea (2%), appetite loss (2%), phlebitis (2%), and alopecia (2%). Most AEs were controllable. G-CSF was administered to one of the three FN patients. The incidence of nonhematological AEs such as arthralgia, myalgia, and peripheral neuropathy was lower during EC therapy than during nab-PTX therapy, suggesting that the AEs caused by nab-PTX resolved in a short period of time, as reported previously [10].

**Table 2:** Most common adverse events

Adverse events	nab-PTX (*n* = 54)			EC (*n* = 52)		
	All grades	Grade 3	Grade 4	All grades	Grade 3	Grade 4
Hematologic						
Leucopoenia	37 (68.5)	4 (7.4)	3 (5.6)	35 (67.3)	12 (23.1)	6 (11.5)
Neutropenia	38 (70.4)	13 (24.1)	13 (24.1)	31 (59.6)	10 (20.4)	15 (28.8)
Febrile neutropenia	0	0	0	4 (7.7)	3 (5.8)	0
Anemia	0	0	0	10 (20.4)	0	0
Thrombocytopenia	4 (7.4)	0	0	3 (5.8)	0	0
AST/ALT increased	20 (37)	3 (5.6)	0	9 (17.3)	1 (1.9)	0
Nonhematologic						
Fatigue	6 (11.1)	0	0	12 (23.1)	1 (1.9)	1 (1.9)
Appetite loss	2 (3.7)	0	0	17 (32.7)	1 (1.9)	1 (1.9)
Nausea/vomiting	0	0	0	30 (57.7)	1 (1.9)	1 (1.9)
Peripheral sensory neuropathy	32 (59.3)	4 (7.4)	0	9 (18.4)	1 (1.9)	1 (1.9)
Peripheral motor neuropathy	6 (11.1)	1 (1.9)	0	1 (1.9)	0	0
Arthralgia	37 (68.5)	8 (14.8)	0	9 (17.3)	0	0
Myalgia	38 (70.4)	7 (13)	0	6 (11.5)	0	0
Phlebitis	0	0	0	2 (3.8)	1 (1.9)	1 (1.9)
Rash	2 (3.7)	1 (1.9)	0	0	0	0

### SPARC expression and response to nab-PTX

We performed IHC for SPARC s in 52 patients whose biopsy samples were available. SPARC expression was observed not only in tumor cells, but also in stromal cells (Fig. 3c). SPARC was expressed in tumor cells in ten of the 53 patients (18.9%) evaluated for SPARC (Table 1) and in stromal cells in 47 of the 53 (88.7%) patients. SPARC expression in tumor cells was not associated with pCR (Table 3). SPARC-positive tumors showed a 35.5% shrinkage after nab-PTX therapy, while SPARC-negative tumors showed a 34.0% shrinkage (not significant; Fig. 3d).

**Table 3:** Relationship between SPARC and pCR

	SPARC expression	pCR	
Tumor cell	Positive 10 (18.9%)	2 (20%)	*p* = 1.00
	Negative 43 (81.1%)	10 (20%)	
Stromal cell	Positive 47 (88.4%)	12 (25.5%)	*p* = 0.32
	Negative 6 (11.6%)	0 (0%)	

### Multivariate analysis

Backward stepwise logistic regression analysis was performed for six factors (age, ER, PgR, HER2, lymph node metastasis, and SPARC) in 52 patients (two with no SPARC and histological assessments were excluded). ER, lymph node metastasis, and SPARC were excluded in estimated model, and there was no significant association of SPARC expression, age, and PgR with the efficacy of nab-PTX. However, HER2 expression was found to be a critical factor for pCR (odds ratio, 5.603; *p* = 0.024; Table 4).

**Table 4:** Multiple logistic regression for evaluation of influencing factors, using backward stepwise method with Akaike’s information criteria (AIC) as variable selection

	OR [95% CI]	*p* value
Variable selection		
Age (≧50/<50)	1.957 [0.427, 9.673]	0.389
PgR (+/−)	0.287 [0.058, 1.192]	0.097
HER2 (+/−)	5.603 [1.356, 28.661]	0.024
ER (+/−)	–	
Lymph node metastasis (+/−)	–	
SPARC (+/−)	–	

## Discussion

This phase II clinical trial evaluated the safety and efficacy of NAC with nab-PTX followed by EC for operable breast cancer and found this regimen to be safe and effective, with an overall pCR rate of 22.2%.

Although several reports have been published, a combination of anthracycline and taxan remains the standard regimen for early/locally advanced breast cancer. The final goal of NAC is to achieve a pCR, which is a factor for a good prognosis, particularly in patients with HER2-positive breast cancer and TNBC. In addition, NAC is used for not only downstaging for BCS, but also in vivo testing for chemosensitivity [1]. Paclitaxel and docetaxel are common taxans used for NAC in patients with breast cancer. However, toxicities are frequent with polyethylated castor oil (Cremophor)-based paclitaxel regimens [25]. Hypersensitivity reactions to docetaxel are also frequent [26]. These factors may worsen the prognosis of patients. Furthermore, unpleasant AEs such as peripheral neuropathy are known to persist for prolonged durations [10].

Nab-PTX is a newly developed albumin-bound form of paclitaxel. This agent is associated with a low incidence of allergies (<1%), requires a shorter time for injection, and does not require corticosteroid premedication. Furthermore, nab-PTX results in quicker recovery from peripheral neuropathy compared with docetaxel [11]. These advantages prompted us to devise a new regimen for NAC in patients with operable breast cancer (PerSeUS-BC01), which comprised the triweekly administration of nab-PTX followed by EC. Our regimen was based on the following speculations. First, a recent study suggested that the administration of taxanes before anthracycline during standard NAC improves the pCR rate for patients with breast cancer [16]. Second, AEs, including peripheral neuropathy, arthralgia, and myalgia, caused by nab-PTX can be completely relieved by the end of the regimen.

The overall pCR (ypT0/Tis ypN0) rate was 22.2% (95% CI 12.0–35.6; *p* = 0.006) in the present study, which was not inferior to those reported in studies on FEC (5-FU, epirubicin, and cyclophosphamide) followed by docetaxel [6] or docetaxel followed by FEC [7]. HER2-positive tumors are particularly sensitive to combination therapy including TZ. Recent reports on NAC with nab-PTX demonstrated a favorable pCR rate ranging from 29 to 37%; this was 49–58% for HER2-positive patients who also received TZ [12–14]. These findings were consistent with ours, despite the smaller number of patients in our study. If the number of HER2-rich patients was higher in the present study, we would have observed a higher pCR rate.

According to previous experimental studies, the intratumoral paclitaxel concentration derived from nab-PTX is higher than that derived from standard paclitaxel regimens, resulting in stronger anti-tumor effects. When nab-PTX enters the circulation, transcytosis across the endothelial barrier is facilitated by the binding of albumin to the gp60 receptor and caveolar transport [27]. On the other hand, SPARC is a key regulator for cellular interaction with the extracellular matrix through binding to structural matrix proteins such as collagen and vitronectin, which are homologous to gp60 [28]. Limited data have indicated that high SPARC expression is associated with a poor prognosis in patients with breast cancer [29–32]. Furthermore, studies with xenograft models have demonstrated that nab-PTX enhances tumor targeting through gp60 by increasing concentration of paclitaxel in the tumors and that caveolae-mediated HER2 and SPARC expression may be useful biomarkers for determining the anti-tumor effectiveness of taxans [15, 33, 34]. In the present clinical trial, tumor shrinkage by nab-PTX therapy was achieved in 88.7% (47/53) patients, while the clinical RR was 59.3% (32/54; Fig. 3b). However, Shao et al. [35] indicated that the response to nab-PTX is independent of SPARC expression in non-small cell lung cancer. On the basis of clinical and preclinical data, we performed IHC to investigate the association between SPARC expression and the response to nab-PTX using biopsy specimens and found that SPARC was expressed in tumor cells in ten of 53 (18.9%) assessed patients, with a 35.5% shrinkage after nab-PTX treatment. However, there was no difference in shrinkage between SPARC-positive and SPARC-negative tumors (34.0% shrinkage) after nab-PTX therapy (Fig. 3c, d). There was no relationship between pCR and SPARC expression. SPARC was also expressed in stromal cells in 47 of the 53 (88.4%) with no significant association with pCR. Although SPARC may increase the PTX concentration in tumor cells (tissue including the microenvironment), our data demonstrated that high SPARC expression in tumor cells did not enhance the effectiveness of nab-PTX. Recent publication from German group showed the similar result to our data, indicating that there is no association between SPARC expression and efficacy by nab-PTX followed by EC [15].

Our tested regimen showed a good safety profile with 85.2% completion rate (46/54) of this regimen. During the nab-PTX regimen, only two patients (3.7%) developed allergic rush. Although arthralgia and myalgia were frequent (Table 3), they lasted for approximately 1 week from the third or fourth day of injection and resolved before the next cycle. Peripheral sensory neuropathy was also completely resolved by the end of chemotherapy.

The results of our study are limited because of the single-arm design, the small sample size, and the lack of a long-term follow-up. Further randomized controlled trials with large sample sizes are necessary. Adjuvant dose-dense doxorubicin and cyclophosphamide followed by dose-dense nab-PTX or weekly nab-PTX may also be feasible in patients with early breast cancer [12, 36]. In addition, combination therapy with anti-HER2 agents including TZ will be more powerful [37, 38]. These factors should be assessed with regard to the effects of neoadjuvant nab-PTX-containing regimens in future.

In conclusion, we demonstrated the effects of a novel preoperative NAC regimen with nab-PTX (plus TZ in HER2-positive patients) followed by EC. The regimen achieved a pCR rate of 22.2% with a good safety profile. Multivariate analysis demonstrated that HER2 is a critical factor for pCR. However, SPARC expression was not associated with pCR and did not affect the efficacy of nab-PTX. Although sensory neuropathy, arthralgia, and myalgia were common AEs after nab-PTX therapy, they were tolerable and resolved by the end of NAC. Therefore, this regimen appears to be an effective alternative for NAC in patients with operable breast cancer. Further studies are necessary to clarify our findings.
